# Mechanism of Action of Prolyl Oligopeptidase (PREP) in Degenerative Brain Diseases: Has Peptidase Activity Only a Modulatory Role on the Interactions of PREP with Proteins?

**DOI:** 10.3389/fnagi.2017.00027

**Published:** 2017-02-14

**Authors:** Pekka T. Männistö, J. Arturo García-Horsman

**Affiliations:** Division of Pharmacology and Pharmacotherapy, Faculty of Pharmacy, University of HelsinkiHelsinki, Finland

**Keywords:** prolyl oligopeptidase, neuropeptides, aging neuroscience, neurodegeneration, proten-protein interactions

## Abstract

In the aging brain, the correct balance of neural transmission and its regulation is of particular significance, and neuropeptides have a significant role. Prolyl oligopeptidase (PREP) is a protein highly expressed in brain, and evidence indicates that it is related to aging and in neurodegenration. Although PREP is regarded as a peptidase, the physiological substrates in the brain have not been defined, and after intense research, the molecular mechanisms where this protein is involved have not been defined. We propose that PREP functions as a regulator of other proteins though peptide gated direct interaction. We speculate that, at least in some processes where PREP has shown to be relevant, the peptidase activity is only a consequence of the interactions, and not the main physiological activity.

## Introduction

Processes leading to the brain aging are complex, and scarcely known. A popular current view is that the housekeeping mechanisms in the cells get faulty and damage starts to accumulate, leading eventually to cell degeneration. An increase of redox stress is important in aging, as are the processes involved in the managing cellular waste, and the maintenance of homeostatic turnover of biological molecules, especially of proteins. In fact, all regulatory mechanisms within cells, among cell-cell communication and those for organ homeostasis are susceptible of aging (Knight, 2000). In the brain, the correct balance and regulation of neural transmission are of particular significance, and neuropeptides play a significant role.

Prolyl oligopeptidase, also known as post-proline cleaving enzyme or prolyl endopeptidase, abbreviated PO, PE, PEP, or POP (here called PREP) was discovered on the early 70's as an oxytocin cleaving enzyme (Walter et al., 1971) and further described as a peptidase able to cleave short peptides (<30 a.a.) with a high specific cleavage at the C-side of proline (Szeltner and Polgar, 2008). A relevant role of PREP in the brain was proposed when a number of neuroactive peptides were described to be its substrates. Early experiments indicated that PREP seemed to be involved in learning and memory and studies on animal amnesia models showed PREP inhibitors to be beneficial but not in all experiments (reviewed in García-Horsman et al., 2007; Männistö et al., 2007). On the other hand, altered PREP activity levels in the brain of neurodegenerative disease patients seemed to be associated with dementia. Furthermore, indications that PREP expression in the brain increases with age were reported (Rampon et al., 2000). Accordingly, search and development of potent and specific inhibitors boomed, several patents were filed, and pharmaceutical companies started clinical trials with PREP inhibitors as memory enhancers for senile- or neurodegeneration- associated dementia (Männistö et al., 2007; Lambeir, 2011b). Despite the intense research on the biological relevance of PREP inhibition, a consensus has not been reached on the identity of the substrate peptides, or on the specific molecular brain pathways where PREP would be exerting its role (Lambeir, 2011b).

The evidence of protein-protein interactions of PREP with α-tubulin, GAP-43, and α-synuclein, independent of peptidase activity (Schulz et al., 2005; Di Daniel et al., 2009; Lambeir, 2011a), has led to the idea that PREP has not only a role in cleaving off physiologically active peptides, but probably also in modulating the function of protein partners.

In this perspective, we propose that the physiological role of PREP results from its direct interaction with partner proteins, which in turn is modulated by peptides and their hydrolysis. We speculate that, at least in some processes where PREP has shown to be relevant, the peptidase activity is a consequence of the protein-protein interactions, and not the main activity. In this paper, we only describe briefly some of the distinct research on PREP in the brain, which we found relevant for this proposal. We do not aim to comprehensively review the recent literature on PREP, and fine studies dealing with peripheral actions of PREP and its role in metabolic-inflammatory diseases, including cancer, are not discussed in this paper.

## PREP expression/regulation in the brain

In the healthy adult brain, PREP is expressed in the cytoplasm of the neurons, at low or high level, with no specific preference in terms of associations with neurotransmitter specific fibers (Myöhänen et al., 2009). PREP levels are particularly high in pyramidal neurons, especially those of the cortical layers II to VI, or those composing the hippocampal CA1 layer. Neuronal populations with hippocampal projections at indusuim griseum and lateral septal area are enriched with PREP. Also, high levels of PREP are present in cerebellar Purkinje cells (Myöhänen et al., 2009). In healthy brain, glial cells are practically devoid of PREP.

It is worth noticing that there is not always a correlation between the protein levels assayed by immunohistochemistry with the PREP peptidase activity measured in enzymatic assays on tissue homogenates (reviewed in Myöhänen et al., 2009). Nevertheless, studies on rodents have shown that the levels of PREP fluctuate in several areas of the brain during embryogenesis and aging, being highest at birth and significantly decreasing in the adult brain (Agirregoitia et al., 2007), but increased again by old age (Rampon et al., 2000). Regulatory mechanisms controlling PREP expression are not known. However, there is evidence that PREP mRNA transcription is controlled by retinoic acid, which support a role of PREP in development (Moreno-Baylach et al., 2011). Furthermore, during neural differentiation *in vitro* (Moreno-Baylach et al., 2008) and *in vivo* (Hannula et al., 2011) PREP is localized within neuronal nuclei of undifferentiated cells, or at early stages of prenatal development. The localization is cytoplasmic in mature neurons, mainly perinuclear and in interaction with cytoskeletal proteins (Schulz et al., 2005). This, combined with the previous discovery that PREP is involved in the regulation of the inositol turnover in several systems (Williams et al., 1999; Schulz et al., 2002), led to the conclusion that there were new intracellular functions of PREP, not related with the extracellular neuropeptide metabolism (Schulz et al., 2005). On the other hand, PREP immunoreactivity (Fukunari et al., 1994), and gene expression (Jiang et al., 2001) have been reported increased in the brains of healthy senescent mice. These findings indicate that PREP participates in age-dependent processes.

In sick or lesioned brain, PREP expression has been shown to be changed. In experimental neuroinflammation, PREP is dramatically overexpressed in glial cells (Penttinen et al., 2011; Tenorio-Laranga et al., 2015), and possibly secreted to the extracellular space. PREP secretion from reactive microglia *in vitro*, has been shown to be toxic to neurons in culture in an inhibitory sensitive manner (Klegeris et al., 2008). On the other hand, in post-mortem analysis of the brains from patients with Alzheimer's or Parkinson's disease, PREP co-localized with pathological plaque deposits (Hannula et al., 2013).

## Effect of PREP inhibitors in behavior, memory and learning

The effects of PREP inhibitors on learning and memory have been extensively studied (reviewed in Männistö et al., 2007; Lambeir, 2011b). JTP-4819 is the best studied PREP inhibitor in the animal models of cognitive functions. It has improved memory in many animal models (Toide et al., 1995, 1997; Shinoda et al., 1997, 1999; Miyazaki et al., 1998), but the effects have rarely been robust or dose-dependent. Another intensively studied PREP inhibitor S-17092 has been even in clinical studies (Morain et al., 2002). Obviously S-17092 has not been promising enough, since the latest reports from the human studies date back to year 2007 (Morain et al., 2007). In the radial-arm maze, a very potent PREP inhibitor, KYP-2047, have shown no effects in scopolamine-treated old and young rats (Peltonen et al., 2010), but their performances were age-dependent; KYP-2047 alone had effects on motility, increasing it in young rats, but decreasing in older ones. On the other hand, in the water maze test (Jalkanen et al., 2007), KYP-2047, dose-dependently improved performance of scopolamine-treated young rats but not in older animals.

It is fair to conclude that the role of PREP in improvement of memory and learning is inconsistent and where an effect has been observed, the underlying mechanisms have not been identified (Männistö et al., 2007). A common theory to explain the role of PREP in memory and learning has been the increase in the levels of cognition-enhancing neuropeptides, such as substance P, TRH, and AVP, by PREP inhibition. However, this has been more like an assumption than a proven fact. The effects of PREP inhibitors on neuropeptide levels have been tested, but the results have varied with the PREP inhibitor used, the length of the treatment and the brain area studied (Männistö et al., 2007). It has been speculated that there are many enzymes participating in the neuropeptide metabolism that blocking one enzyme, like PREP, may not be sufficient to increase the levels of neuropeptides (Jalkanen et al., 2007). Therefore, there are no strong experimental evidence supporting that PREP would participate in cognitive, or other behavioral processes, through neuropeptide metabolism.

In other experiments, lesioning the dopaminergic medial forebrain bundle by 6-OHDA did not affect the activity or expression of PREP in the striatum and PREP inhibitors had no effect (Peltonen, 2012). However, the single i.p. treatment with KYP-2047 decreased the levels of dopamine in the striatum and increased the levels of dopamine metabolites in the VTA (Myöhänen et al., 2007). A recent study, found changes in the levels of phosphorylated dopamine transporter (DAT) in mice, dependent on PREP levels of expression (Julku et al., 2016). The data suggests that PREP modulates DAT internalization, which in turn modifies extracellular dopamine levels. The mechanism of this regulation is not known, but the study did not investigate changes on any specific neuropeptide (Julku et al., 2016).

## Behavior of PREP knock-out mice

Di Daniel et al. (2009) demonstrated a disturbed control of growth cone and synaptic function in a full PREP knock out mice. These mice were aggressive and hyperactive but no other behavioral findings were reported. A gene trap (GT) mouse with PREP significantly knocked down was reported by Warden et al. (2009). Interestingly, this study reported a decrease on α-melanocyte-stimulating hormone levels; opposite of what it would have been predicted if this peptide was a substrate of PREP. Also in this work, an evidence was found of disruption of processes where vasopressin and oxytocin are involved, but no evidence of changes of the levels of these peptides was reported. Using the same GT strain, D'agostino et al. (2013), detected a decrease of long term potentiation in GT animals consistent with the spatial memory impairment in the Morris water maze test. A more detailed behavioral phenotyping of the PREP deficiency seems to affect mouse motor activity and anxiety-like behavior (Höfling et al., 2016).

Other studies on PREP-KO mice showed association of PREP and neuroplasticity modulation probably mediated by inflammatory response where polysialylated-neural cell adhesion molecule (PSA-NCAM) levels are altered in the brain (Höfling et al., 2016). Recent findings, using PREP overexpressing neuroblastoma cells, indicate that PREP is implicated in the regulation of PS-NCAM, most probably by modulating the proteases which normally degrade NCAM forms (Jaako et al., 2016).

## PREP in psychiatric disorders

There are several reports describing fluctuations on PREP levels in patients suffering from depression, anxiety and bipolar disorder (for reviews see e.g., García-Horsman et al., 2007; Lambeir, 2011b). However, no peptides, which would link PREP with those disorders have been identified. The most notable finding in this area is the opposite action of PREP and lithium, a well-known mood stabilizer, showing that that PREP is involved in the regulation of inositol signaling, interacting with some of the enzymes which control the levels of neuronal inositol (Williams et al., 1999; Harwood, 2011). Again, no peptide substrate of PREP has been related to any of these findings. This fact could be taken as an argument of ignorance. However, in view that the search for these peptides has been actively pursued, it also stimulates the thinking to other directions. A lack of correlation between neuropeptide levels, behavior and PREP inhibition, described above, adds fuel to the fire.

## Enzymatic activity

PREP has endopeptidase activity. It cleaves short peptides at the carboxyl side of proline, with the exception of the prolyl-prolyl bond. There are few cases where PREP cleaves (*in vitro*) after alanine and cysteine, but with much lower efficiency (Polgar, 1992; Szeltner et al., 2002a; Bar et al., 2006).

There are many proline containing biologically active peptides, often neuropeptides. Because proline is the only amino acid with a secondary amine group, it confers special secondary structure to peptides, and its presence in the peptide chain confers resistance to degradation by most proteases. Thus, PREP has been considered to be the specific hydrolyser of proline-containing neuropeptides. In fact, any short proline-containing peptide will be degraded by PREP *in vitro*, and indeed, there are dozens of peptides reported to be substrates of PREP (for reviews see García-Horsman et al., 2007; Brandt et al., 2007). Table 1 summarizes the data on the few neuropeptides where more extensive research has been conducted on relation to PREP activity. In all, a validation of PREP being responsible for the metabolism of those peptides *in vivo* has not been conclusive. There have been fine efforts to identify the physiological substrates of PREP in the brain, using sophisticated mass spectrometric techniques on hypothesisless experimental approaches (Brandt et al., 2005; Nolte et al., 2009; Tenorio-Laranga et al., 2009, 2011, 2012; Lone et al., 2010). However, the results have been scanty. These findings, combined with the inconsistency of those obtained from *in vivo* behavioral data described above, rise concern on the physiological relevance of the peptidase activity of PREP.

**Table 1:** **Most researched peptides as substrates of PREP in the brain and the evidence *in vivo* and *in vitro***.

	**Evidence of physiological PREP digestion**	
**Neuropeptide**	***in vivo***	***in vitro***
Substance P (SP)	Levels of SP, or peptide derivatives, in brain after *in vivo* PREP inhibition, have been found changed^a^ or unchanged^b^. Increase of SP immunoreactivity levels after PREP inhibition *in vivo*^c^. No changes of SP in rat striatum by *in vivo* microdialysis^d^. Potentiation^e^ of SP activity.	SP degradation sensitive to specific PREP inhibitors in tissue homogenates^f^. Evidence that SP is degraded by fibroblast activation protein α^g^. No immuno co-localization of PREP with SP, or NK-1 receptors^h^.
Thymosin β_4_ (TB4)	TB4 fragments (Ac-SKDP) levels altered in whole animals^a^. Inhibition of Ac-SDKP action in whole animals by PREP inhibition^i^.	Increase on Ac-SKDP release from TB4 upon^i^.
Thyrotropin releasing hormone (TRH)	PREP inhibitors increase TRH immunoreactivity in some areas of the rat brain^j^. PREP inhibitors did not changed effect of administrated TRH^k^.	Increased or unchanged TRH immunoreactivity upon PREP inhibitors^l^.
Gonadotropin releasing hormone (GnRH)	No *in vivo* evidence^l^.	PREP inhibitor sensitive *in vitro* degradation of GnRH in tissue homogenates^l^.

a *Nolte et al. (2009)*.

b *Tenorio-Laranga et al. (2009, 2012)*.

c *Bellemère et al. (2003)*.

d *Jalkanen et al. (2011)*.

e *Schulz et al. (2002)*.

f *Saidi et al. (2016)*.

g *Keane et al. (2011)*.

h *Myöhänen et al. (2008)*.

i *Myöhänen et al. (2011)*.

j *Bellemère et al. (2005)*.

k *Lazcano et al. (2012)*.

l *Reviewed in García-Horsman et al. (2007)*.

Compared with other proteases, the enzymatic peptide cleavage activity of PREP is relatively poor. Depending on the peptide substrate, the turnover numbers of mammalian PREP are around one per second, and the catalytic efficiencies are below 1 M^−1^ per second; the Km for substrates are in the order of 10^−4^ M or higher (METROPS the peptidase database, http://merops.sanger.ac.uk/; Rawlings et al., 2016). At physiological conditions, peptide concentrations are usually much below those levels, and then the cleavage of the peptides would be much slower. Moreover, and to complicate the scenario, endogenous systems which inhibit PREP have been identified (Tenorio-Laranga et al., 2013). Thus, the peptide substrates would have to compete with them to be efficiently hydrolysed by PREP. Notably, the levels of protein expression of PREP seldom correlates with the activity levels or with its mRNA levels. This strongly suggests that there are processes controlling PREP's transcription, translation, and enzymatic activity, and the actual modulation in PREP protein levels might not be meant to alter the peptidase activity. Finally, PREP is a large protein if it is considered to have only the protease activity.

## Protein-protein interactions, PPIs

In earlier experiments, a particular intracellular distribution of PREP was described (Rossner et al., 2005; Schulz et al., 2005), which prompted to propose a role of PREP in protein secretion and/or axonal transport within the neurons, probably through tight interactions with structural proteins (Table 2). In fact, an evidence of a direct interaction of PREP with α-tubulin was found (Schulz et al., 2005). Consistent with this proposal, further experiments with PREP-null mutations found altered growth cone dynamics (Di Daniel et al., 2009). These alterations were reversed in the PREP-null cells by virally expressed wild type PREP. Surprisingly, a full reconstitution of activity was observed also when the cells were transfected with a catalytically inactive PREP mutant. That study also showed an interaction of PREP with growth associated protein 43 (GAP-43), a regulator of neural plasticity.

**Table 2:** **Proteins that have protein interactions with PREP**.

**Protein**	**Evidence**	**References**
α-tubulin	Detected by two-hybrid screen. Immunohistochemical colocalization.	Schulz et al., 2005
α-synuclein	Inferred by α-synuclein anti-aggregation effect of PREP. Detected by protein-fragment complementation assays and microscale thermophoresis	Van der Veken et al., 2012; Lambeir, 2011a; Savolainen et al., 2015
GAP-43	Interaction by yeast-two-hybrid, co-precipitation and ELISA assays	Di Daniel et al., 2009; Szeltner et al., 2011
α-2-macroglobulin	Co-purification	Tenorio-Laranga et al., 2013

It has been reported that a POP inhibition suppressed the mRNA levels of glyceraldehyde-3-phosphate dehydrogenase (GAPDH), decreased age-induced apoptosis and also prevented GAPDH nuclear translocation in cultured neurons (Katsube et al., 1999). Effects on GAPDH translocation was also found in fibroblasts (Puttonen et al., 2006). Another study described physical interaction of PREP with GAPDH, and suggested that this would modulate GAPDH translocation to the nucleus during apoptosis in neuroblastoma cells (Matsuda et al., 2013).

The most studied interaction of PREP is with α-synuclein (Di Daniel et al., 2009), reviewed by Lambeir (2011a). Brandt et al. (2008), found that PREP was able to interfere with the aggregation process of α-synuclein *in vitro*. In those experiments, it was relevant that the stimulation of the aggregation of α-synuclein by PREP was not due to peptide cleavage, but it was occurring independent of PREP activity by a process that was anyhow sensitive to PREP inhibitors, since catalytically inactive mutants of PREP showed same effect. The interaction of PREP with α-synuclein aggregates was also observed in cellular models and in *in vivo* mouse models over-expressing α-synuclein (Myöhänen et al., 2012; Savolainen et al., 2014). The mechanism of how PREP inhibitors are promoting α-synuclein disaggregation is not known, but it certainly seems not to be due to the inhibition of peptide cleavage activity of PREP. Rather it happens by modulating PPIs with α-synuclein, or other partners, probably in a chaperone-like process.

As mentioned, it has been proposed that PREP might be involved in the protein secretion (Schulz et al., 2005; Morawski et al., 2011). Indications that PREP participates in the secretion of hormones that control glucose homeostasis have been reported (Kim et al., 2014). Secretion, and protein clearance mechanisms have been explored to try to define the process where PREP inhibitors may be involved in decreasing α-synuclein aggregation in the brain. The results indicate that PREP might be involved in the control of autophagy (Savolainen et al., 2014), by still unknown mechanisms, without indications of specific hydrolysis of a peptide substrate of PREP.

## Protein structure/molecular modeling

**Figure 1:**
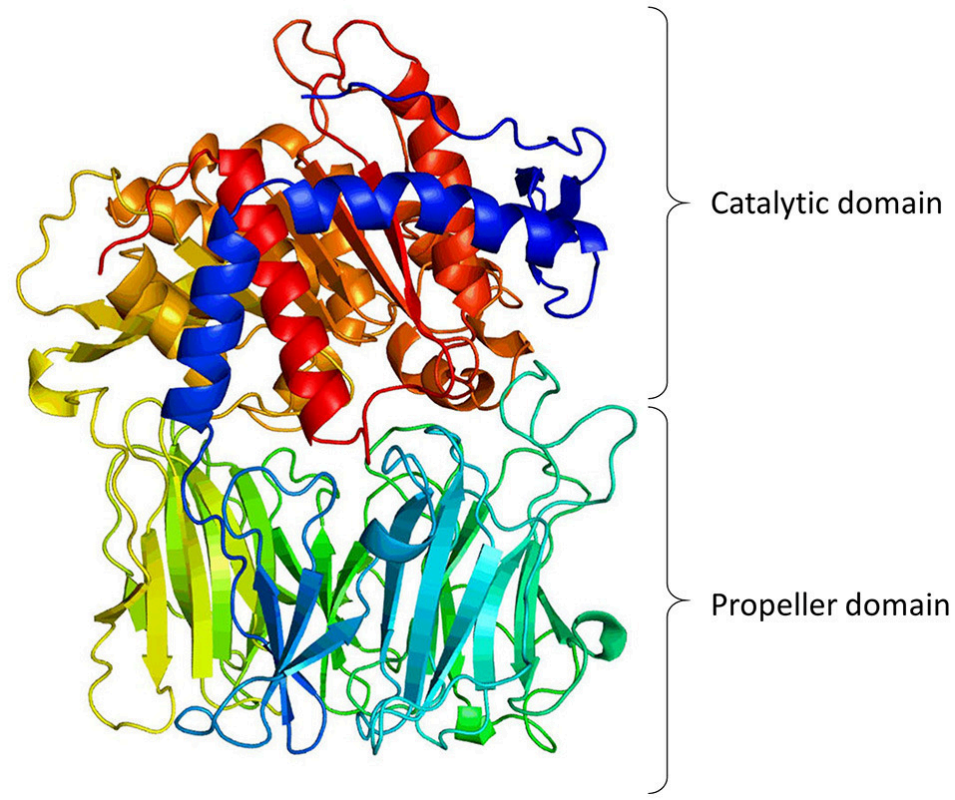
**3-Dimentional model of porcine PREP based on its crystal structure (Fülöp et al., 1998, 2001; Rea and Fülöp, 2011)**. The figure shows the catalytic domain (α/β-hydrolase fold) and the propeller domain (7-bladed β-propeller fold).

Structural information indicates that PREP has two distinctive structural components (Figure 1): the protease catalytic domain, with a classic α-β hydrolase fold (residues 1–71 and 436–710), and a seven-bladed β-propeller domain (residues 72–435). When the crystal structure of PREP was solved (Fülöp et al., 1998), it seemed evident that the propeller domain might function as a substrate gate. Very detailed kinetic studies supported this, showing that the propeller may acts as a gating filter, allowing the access of only short peptides to the active site (Fülöp et al., 2000; Szeltner et al., 2000; Szeltner and Polgar, 2008). Molecular dynamic studies have predicted an important role of the β-propeller in substrate gating, but they also have shown that the domain experience conformational changes that expose or hide certain residues upon ligand binding (Kaushik and Sowdhamini, 2011; Kaszuba et al., 2012; Lopez et al., 2016; Van Elzen et al., 2017). Further studies have indicated that PREP seems to exist naturally in three conformations, with different catalytic and PPIs features, in which the loops at the interface of both catalytic and propeller domains are crucial components (Szeltner et al., 2011, 2013).

The β-propeller folding is present in a large variety of proteins, and in most cases the function of this domain is to mediate PPIs (Chen et al., 2011). Therefore, it is feasible to think that the main function of this domain in PREP is to interact with large proteins. Molecular dynamics of PREP has revealed that ligand binding modifies the protein dynamics of the domain interface, probably favoring one of the three conformations (Szeltner et al., 2011; Kaszuba et al., 2012; Lopez et al., 2016).

**Figure 2:**
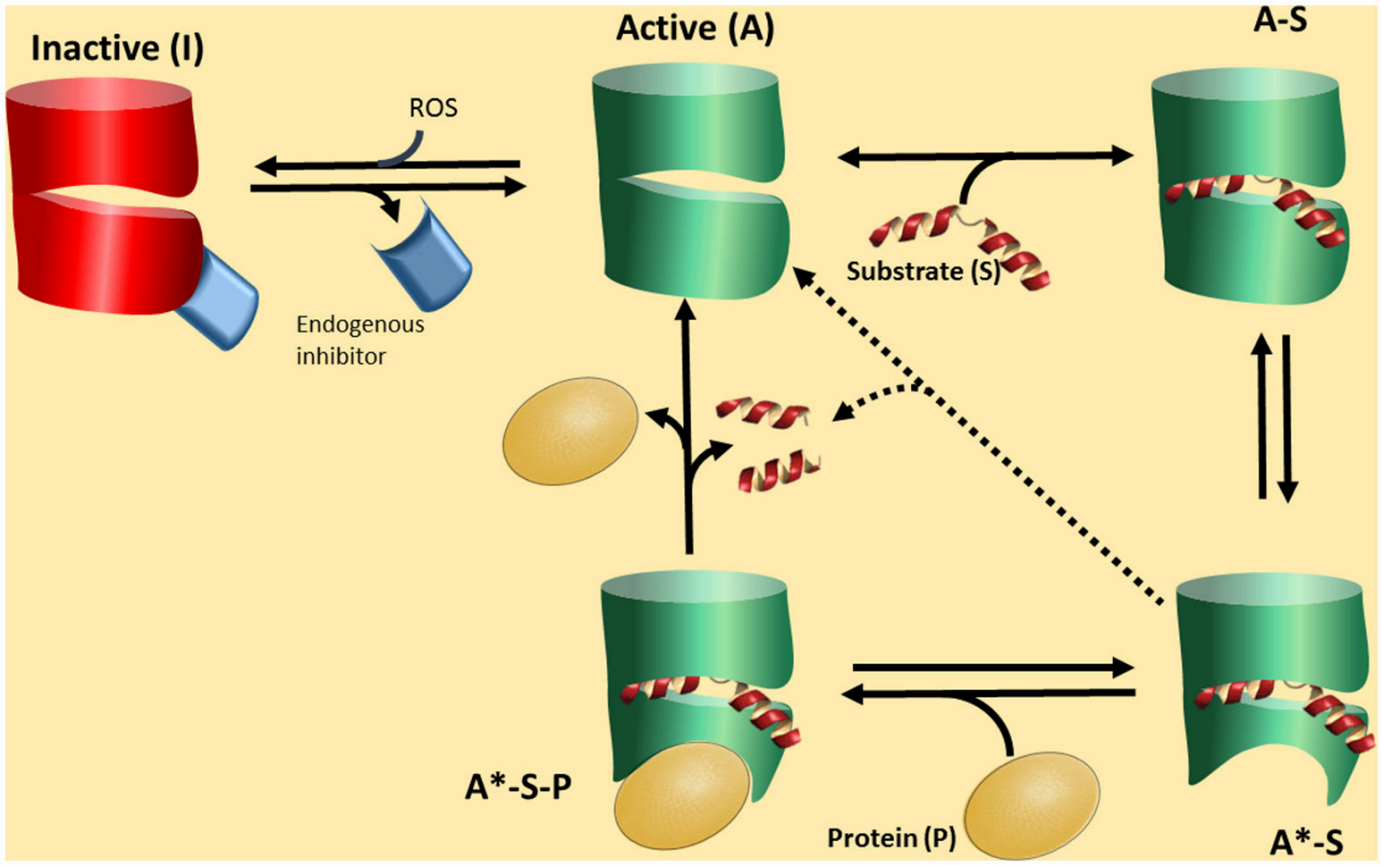
**A proposed functional cycle for PREP. PREP in an active form (A) can bind a substrate peptide (S) to become the form A-S**. This form is prone to produce a conformational change in PREP, depicted as a red area, becoming A^*^-S. This is a stable complex driving the equilibrium A to A-S to the formation of A-S. The A^*^-S form, due to the particular conformation, is now able to interact with a protein partner (P) forming a tertiary complex A^*^-S-P, which is the biologically active form. The tertiary complex A^*^-S-P can be broken by substrate cleavage, regenerating the form A, with low affinity by the partner P. The form active A can be deactivated by interaction with an endogenous inhibitor (EI) or/and reactive oxidative species (ROS). The form A^*^-S could slowly lead to substrate cleavage.

All this information along with the fact that there have been no definitive conclusions on “the substrates” of PREP, a different hypothesis could be presented on the function of PREP. Shortly: PREP interacts with a protein partners, and the strength of the interaction will depend on the conformation of PREP. This conformation will be in turn mainly dictated by the presence of a ligand, a peptide, and the control of this switch will be mediated by cleavage of the peptide (Figure 2).

In the Figure 2 we propose a scheme of this novel scenario. PREP would interconvert among three basic conformations. The closed form I is inactive and closed to ligand/peptide binding. Stabilization of this form is caused at least by two factors. One is the redox environment. It has been recognized that PREP responds to redox conditions so that reductants activate the enzyme (Szeltner et al., 2002b, 2003; Agustí-Cobos and Tenorio-Laranga, 2011). Furthermore, redox conditions also change the interaction with lipids and other partners (Tenorio-Laranga et al., 2008; Szeltner et al., 2011, 2013). The second is an endogenous inhibitor (Figure 2). Such inhibitors have indeed been described in the literature (Soeda et al., 1986; Salers, 1994; Tenorio-Laranga et al., 2010, 2013; Telford et al., 2011). The open, or active form may exist in different species: form A is open when empty, and the population of this species is probably very low, especially when appropriated concentrations of the peptide substrate (S) are present and tightly bound to PREP to produce the form A-S. The A-S form may also be a very transient species since it turns to the form A^*^-S, which has a conformational change around the propeller region, induced by the ligand (substrate or inhibitor) binding. Conformational changes in this domain, upon ligand binding, have been demonstrated (Szeltner et al., 2000, 2004; Kiss et al., 2004). We suggest that the form A^*^-S is able to interact with a PPI partner (P in Figure 2), and subsequently form a stable tertiary complex, the form A^*^-S-P. This form may have a particular biological activity which prevails as long as it is stable. Its stability may be broken by the hydrolysis of the peptide by PREP. PREP inhibitors may have different activities. One may stabilize the PPI active forms of PREP (A^*^-S, and in turn also A^*^-S-P), or stabilize the form A-S, which is not able to interact with the PPI partner. Fine kinetic studies have indicated that PREP inhibitors show a complex kinetics, and allosteric-like interaction have been proposed (Fülöp et al., 2001; Szeltner et al., 2004; Fuxreiter et al., 2005; Juhasz et al., 2005). In the absence of a PPI partner, e.g., *in vitro*, the cycle could short circuit from form A^*^-S (or A-S) to form A, through a catalytic cleavage by peptidase, but this is predicted to be a slow process. The relative low activity of PREP *in vitro*, compared with other peptidases, support this notion. In fact, despite appreciable amounts detected in circulation, some researchers are not able to detect any activity at all (Lee et al., 2011). In this cycle, we would like to point that it is the formation of a complex PREP-Partner (form A^*^-S-P) which might be physiologically relevant, and peptide binding and cleavage, are only regulatory factors.

It is interesting to note that PREP-like protein (PREPL), a member of prolyl oligopeptidase family, has a structure highly homologous to PREP (Szeltner et al., 2005) including a β-propeller and a catalytic domain. Even if PREPL is catalytically active, no peptides/proteins have been found to be cleaved by it, in spite of the great efforts to find them (Martens et al., 2006; Boonen et al., 2011). PREPL deletion produces severe growth impairment (Lone et al., 2014), and indeed it has been speculated that its function is to interact with other proteins important in growth, in a similar fashion to PREP (Boonen et al., 2011), probably through a mechanism here proposed. In fact, the intracellular localizations of PREPL and PREP are very similar (Morawski et al., 2013), which has led to the suggestion that both proteins have a redundant function. On the other hand, similar PPI function mechanism could be speculated for two enzymatically inactive dipeptidyl peptidases DPP6 and DPP10. Although structurally less homologous to PREP than PREPL, they are implicated in a number of neuronal processes in health and disease (McNicholas et al., 2009), and ligands (peptides?) may be proposed to regulate their PPI activities even in the absence of an active center.

## Conclusions

PREP has been implicated in several of brain processes, like memory, learning, secretion, plasticity, and aging. Since PREP has an enzymatic activity for cleaving short peptides, it has been logically related in regulation of the neuropeptide levels (García-Horsman et al., 2007; Männistö et al., 2007; Lambeir, 2011b). However, extensive research has not been able to definitively identify the physiological substrate peptides. Potent and selective PREP inhibitors have not robustly elevated the putative substrate levels, and even inactive PREP mutants have retained some functions, suggesting that peptidase activity is evidently not always required for some of the processes where PREP has been associated to. We here propose that rather than the peptidase activity, it is the direct PREP-other protein interaction that is primarily involved in the several processes mentioned (Figure 2). We suggest that PREP regulates through this interaction the activity of other proteins such as proteases, transport proteins and chaperones, according to the biological conditions and type of the cell in question, and according to the prevalent peptide homeostasis. Enzymatic activity of PREP may act only as a regulator of its interactions with other proteins.

Aging is a complicated process where both genes and environment play key roles and interact.

However, the mechanisms of how these factors influence longevity is still not understood. There is some evidence that cellular systems of housekeeping, repair and stress responses are crucial. Regulation of these processes is still mostly unknown. New hypothesis may be a useful starting point to fill the gaps.

## Author contributions

JG developed the idea. PM participated in the discussion of the idea. JG and PM wrote the manuscript.

### Conflict of interest statement

The authors declare that the research was conducted in the absence of any commercial or financial relationships that could be construed as a potential conflict of interest.
